# Crystal structure of 3-{1′-[3,5-bis­(tri­fluoro­meth­yl)phen­yl]ferrocenyl}-4-bromo­thio­phene

**DOI:** 10.1107/S1600536814020674

**Published:** 2014-09-24

**Authors:** Elisabeth A. Poppitz, Marcus Korb, Heinrich Lang

**Affiliations:** a Technische Universität Chemnitz, Fakultät für Naturwissenschaften, Institut für Chemie, Anorganische Chemie, D-09107 Chemnitz

**Keywords:** crystal structure, ferrocenyl backbone, thio­phene, Negishi cross-coupling

## Abstract

The mol­ecular structure of the title compound, [Fe(C_9_H_6_BrS)(C_13_H_7_F_6_)], consists of a ferrocene backbone with a bis­(tri­fluoro­meth­yl)phenyl group at one cyclo­penta­dienyl ring and a thio­phene heterocycle at the other cyclo­penta­dienyl ring. The latter is disordered over two sets of sites in a 0.6:0.4 ratio. In the crystal structure, intra­molecular π–π inter­actions between the thienyl and the phenyl substituent [centroid–centroid distance 3.695 (4) Å] and additional weak *T*-shaped π–π inter­actions between the thienyl and the phenyl-substituted cyclo­penta­dienyl ring [4.688 (6) Å] consolidate the crystal packing.

## Abstract 2

The first five-membered group-VI 3-ferrocenyl heterocycle bearing a further aromatic substituent at the 1′ position of the ferrocene backbone is reported.

## Chemical context   

The use of ferrocenyl (Fc) functionalized thio­phenes as redox-active metal-based monomers offers the possibility of designing new conductive materials, such as polymers and mol­ecular wires (see, for example: MacDiarmid *et al.*, 2001 ▶; Barsch *et al.*, 1994 ▶; Heeger *et al.*, 2001 ▶; Speck *et al.*, 2012 ▶; Pfaff *et al.*, 2013 ▶; Hildebrandt *et al.*, 2011 ▶; Hildebrandt & Lang, 2013 ▶; Wolf, 2001 ▶; Zhu & Wolf, 2000 ▶; Zotti *et al.*, 1995 ▶). The electrochemical inter­action between the thio­phene donor and the ferrocenyl acceptor with different conjugated 2-Fc—C C-(5-^c^C_4_H_2_S)_*n*_(^c^C_4_H_3_S) (*n* = 0, 1, 2), 2-Fc—C C-[5-(3,4-OCH_2_CH_2_O)(^c^C_4_S)]_*n*_(3,4-OCH_2_CH_2_O)^c^C_4_HS (*n* = 0, 1, 2) and 2,5-(Fc—C C)_2_-(^c^C_4_H_2_S)_*n*_ (*n* = 1, 2, 3), 2,5-(Fc—C C)_2_-[(3,4-OCH_2_CH_2_O)(^c^C_4_S)]_*n*_ (*n* = 1, 2, 3) were studied by Zhu & Wolf (1999 ▶). The results of the spectro- and electrochemical measurements showed an inter­esting insight into the conductibility, which may lead to an improvement of sensor technology using conductive polymers. Electron-withdrawing and donating groups on the ferrocenyl or the thienyl moieties have been used to modify the charge-transfer properties. This has been shown for a series of different 2,5-diferrocenyl thio­phenes (Speck *et al.*, 2014 ▶). In continuation of this work, we present herein the synthesis and crystal structure of 3-{1′-[3,5-bis­(tri­fluoro­meth­yl)phen­yl]-1,1′-ferrocenedi­yl}-4-bromo­thio­phene, [Fe((C_9_H_6_BrS)C_13_H_7_F_6_)], (I). The synthesis of this compound was realized using typical Negishi *C,C*-cross-coupling reaction conditions.
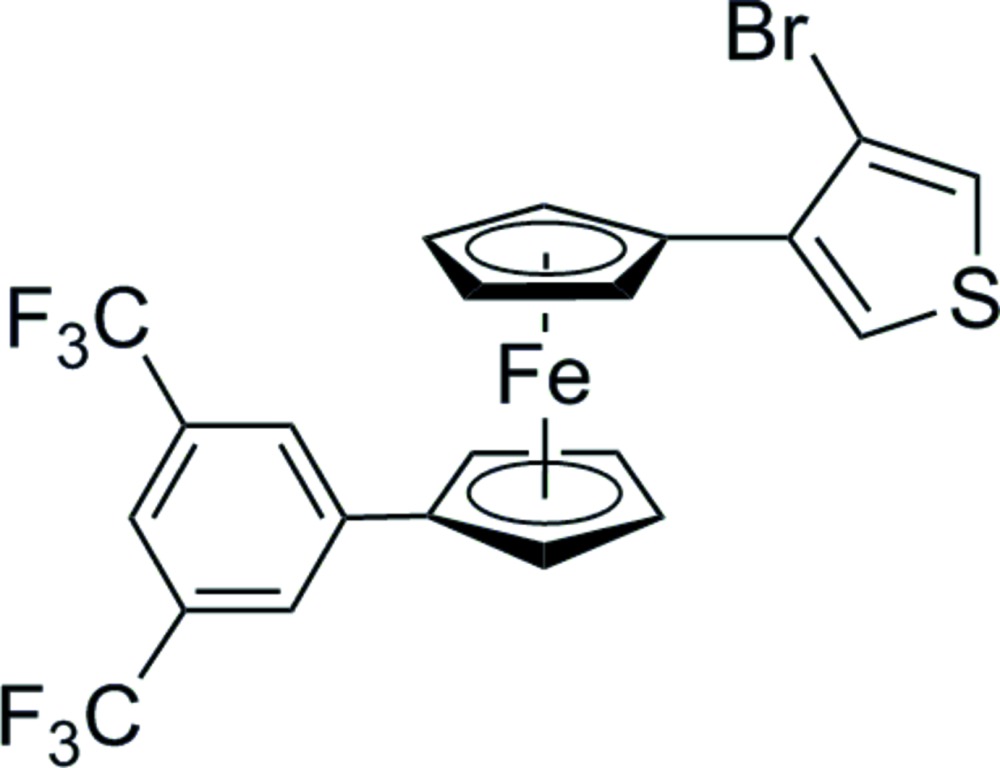


## Structural commentary   

**Figure 1:**
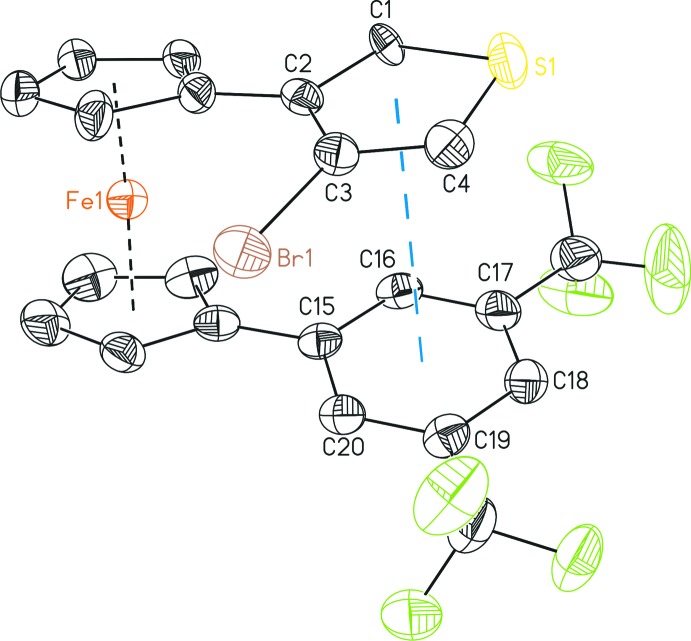
The mol­ecular structure of (I) showing short intra­molecular π–π inter­actions between the thienyl and the phenyl substituents, with displacement ellipsoids drawn at the 50% probability level. All hydrogen atoms, the minor disordered part of the structure and further π–π inter­actions have been omitted for clarity.

The title compound contains one mol­ecule in the asymmetric unit with an intra­molecular π–π distance between the centroids (*D*) of the thio­phene and the phenyl substituents (Fig. 1 ▶) of 3.695 (4) Å (Table 1 ▶) (Sinnokrot *et al.*, 2002 ▶) favoured by the nearly coplanar cylo­penta­dienyl rings [*D*(C_5_H_4_)—Fe—*D*(C_5_H_4_): 175.84 (3) and 175.66 (3)°] in the ferrocenyl backbone. For the disordered part (′-labeled, see: *Refinement* and additional Figure in the supporting information), however, the distance of 3.871 (6) Å is too long for a π–π inter­action caused by the increased torsion angle between the substituents in the 1- and 1′-position [9.2 (4)° for the main part; 16.7 (5)° for the disordered part]. The mean planes of the cyclo­penta­dienyl rings and the bonded aromatic rings are almost coplanar with each other [C_6_H_3_—C_5_H_4_, 16.2 (3)°; C_4_H_3_S—C_5_H_4_, 17.3 (6) (main part) and 16.9 (10)° (other part)] and thus, a nearly parallel arranged stacking between the phenyl and the thio­phene rings [8.9 (3)° for the main part and 9.7 (6)° for the other part] is realized.

## Supra­molecular features   

**Figure 2:**
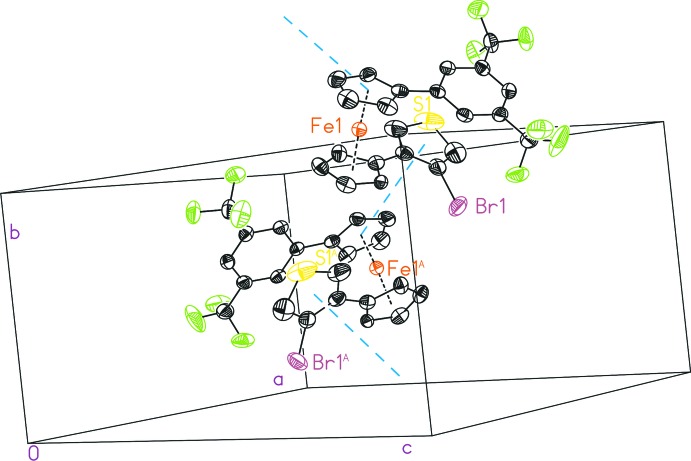
Inter­molecular *T*-shaped π–π inter­actions between the thienyl and the phenyl-substituted cyclo­penta­dienyl rings, with displacement ellipsoids drawn at the 50% probability level. All hydrogen atoms, the minor disordered part of the structure and further π–π distances have been omitted for clarity. [Symmetry code: (A) − *x* + 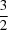, *y* − 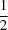, −*z* + 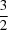.]

Inter­molecular *T*-shaped π–π inter­actions between the thienyl and the phenyl-substituted cyclo­penta­dienyl rings (Fig. 2 ▶) are observed. The disordered part (labeled with ′) exhibits a stronger inter­action of 4.688 (6) Å; in contrast, it is 4.943 (4) Å for the other disordered part, which is rather weak (Table 1 ▶).

## Database survey   

The only reported examples of 3-ferrocenyl-substituted five-membered group-VI heterocycles (Speck *et al.*, 2012 ▶; Hildebrandt *et al.*, 2011 ▶; Claus *et al.*, 2011 ▶) exhibit a similar co-planarity between non-sterically hindered thio­phenes and the cyclo­penta­dienyl rings [10.4 (2)°, Speck *et al.*, 2012 ▶; −6.4 (4)°, Claus *et al.*, 2011 ▶], but a high distortion for thio­phenes bearing further *ortho*-substituents [40.1 (9) to 56.6 (9)°, Speck *et al.*, 2012 ▶; 70.9 (3) and 42.7 (3)°, Hildebrandt *et al.*, 2011 ▶]. The conformations of reported ferrocene derivatives bearing aromatic substituents in the 1 and 1′ positions range from anti­periplanar [180.0 (4), plane twisting 13.99 (15)°, Braga *et al.*, 2003 ▶] and anti­clinal [147.02 (14), plane twisting 33.7 (9)°, Deck *et al.*, 2004 ▶] to synperiplanar [0.3 (3)°, Deck *et al.*, 2000 ▶; −0.5 (9)°, Blanchard *et al.*, 2000 ▶; 4.09 (19)°, Gallagher *et al.*, 2010 ▶; −6.5 (6)°, Hursthouse *et al.*, 2003 ▶; 14.4 (8)°, Foxman *et al.*, 1991 ▶] with plane twists from 12.8 (9) (Gallagher *et al.*, 2010 ▶) to 82.8 (4)° (Foxman *et al.*, 1993 ▶). Furthermore, for all synperiplanar examples, intra­molecular inter­actions between the aromatic planes are present with distances smaller than 3.42 Å (Hursthouse *et al.*, 2003 ▶).

## Synthesis and crystallization   

1-Bromo-1′-(3,5-bis­(tri­fluoro­meth­yl)phen­yl)ferrocene was prepared according to synthetic methodologies reported by Speck *et al.* (2014 ▶). The synthesis of ferrocenyl thio­phene (I) was realized using typical Negishi *C,C*-cross-coupling conditions by reacting 1-bromo-1′-(3,5-bis­(tri­fluoro­meth­yl)phen­yl)ferrocene with 3,4-di­bromo­thio­phene (Negishi *et al.*, 1977 ▶).

Synthesis of (I): For the Negishi *C,C*-cross-coupling reaction, 1-bromo-1′-(3,5-bis­(tri­fluoro­meth­yl)phen­yl)ferrocene (1.0 g, 2.10 mmol) was dissolved in 50 ml of tetra­hydro­furan (THF) and 1.2 equivalents (0.9 ml, 2.52 mmol) of a 2.5 *M* solution of *n*-butyl­lithium in *n*-hexane were added dropwise at 193 K. After 1 h of stirring at this temperature, 1.2 equivalents (0.71 g, 2.52 mmol) of [ZnCl_2_·2THF] were added in a single portion. The reaction was kept for 10 min at this temperature and was then allowed to warm to 273 K during an additional hour. Afterwards, 0.25 mol% of [P(*t*-C_4_H_9_)_2_C(CH_3_)_2_CH_2_Pd(*μ*-Cl)]_2_ and 1.5 equivalents (0.76 g, 3.15 mmol) of 3,4-di­bromo­thio­phene were added in a single portion. The resulting mixture was stirred for 10 h at 323 K. After evaporation of all volatiles, the crude product was dissolved in 30 ml of di­chloro­methane and was washed twice with 50 ml portions of water. The organic phase was dried over MgSO_4_ and the solvent was removed with a rotary evaporator. The remaining orange solid was purified by column chromatography on silica gel using a *n*-hexa­ne/diethyl ether 1/1 (*v*/*v*) mixture. Red crystals of (I) were obtained by slow evaporation of a saturated *n*-hexa­ne/methanol 1/5 (*v*/*v*) solution at ambient temperature. Yield: 660 mg (1.18 mmol, 56% based on 1-bromo-1′-(3,5-bis­(tri­fluoro­meth­yl)phen­yl)ferrocene). IR (KBr, cm^−1^): ν = 1275 (*s*, C—F), 1504 (*s*, C=C), 1615 (*m*, C=C) 2848, 3095 (*w*, C—H). ^1^H NMR (500.3 MHz, CDCl_3_, 298 K, ppm): δ = 7.61 (*s*, 3H, C_8_H_3_F_6_), 7.09 (*d*, 1H, *J*_H,H_ = 3.6 Hz, C_4_H_2_S), 6.90 (*d*, 1H, *J*_H,H_ = 3.6 Hz, C_4_H_2_S), 4.73 (*pt*, 2H, *J*_H,H_ = 1.9 Hz, C_5_H_4_), 4.69 (*pt*, 2H, *J*_H,H_ = 1.9 Hz, C_5_H_4_), 4.46 (*pt*, 2H, *J*_H,H_ = 1.9 Hz, C_5_H_4_), 4.25 (*pt*, 2H, *J*_H,H_ = 1.9 Hz, C_5_H_4_). ^13^C{^1^H} NMR (125.7 MHz, CDCl_3_, 298 K, ppm): δ = 140.64 (*s*, C_*i*_-C_6_H_3_), 135.56 (*s*, C_*i*_-C_4_H_2_S), 131.54 (*q*, *J*_C,F_ = 33 Hz, C_*i*_-C_6_H_3_), 125,63 (*m*, C_6_H_3_), 124.88 (*s*, C_4_H_2_S), 123.50 (*q*, *J*_C,F_ =273 Hz, CF_3_), 121.12 (*s*, C_4_H_2_S), 119.05 (*m*, C_6_H_3_), 109.78 (*s*, C_*i*_-C_4_H_2_S), 82.98 (*s*, C_*i*_-C_5_H_4_), 82.01 (*s*, C_*i*_-C_5_H_4_), 71.40 (*s*, C_5_H_4_), 70.17 (*s*, C_5_H_4_), 68.81 (*s*, C_5_H_4_), 68.20 (*s*, C_5_H_4_). HRMS (ESI–TOF, *M* ^+^): C_23_H_16_F_6_FeSO: *m*/*z* = 557.9291 (calc. 557.9171).

## Refinement details   

Crystal data, data collection and structure refinement details are summarized in Table 2 ▶. C-bonded hydrogen atoms were placed in calculated positions and constrained to ride on their parent atoms with *U*_iso_(H) = 1.2*U*_eq_(C) and a C—H distance of 0.93 Å for the aromatic protons. The thienyl and the attached cyclo­penta­dienyl ring were refined as disordered over two sets of sites with occupancies of 0.6 and 0.4. The spatial proximity of the sulfur and the bromine atom of the disordered part required DFIX [C1—C2 1.51 (2), C2—C3 1.33 (2), C3—C4 1.35 (2) S1—C1 1.62 (2), S1—C4 1.82 (2), C3—Br1 1.94 (2) Å) and DANG (C4—Br1 2.75 (4), C1—C3 2.27 (4), C2—C4 2.38 (4), C4—Br1 2.75 (4) Å] instructions, which were used for the minor disordered part (′-labeled). For both disordered parts, some anisotropic displacement ellipsoids were rather elongated and hence SIMU/ISOR restraints (McArdle, 1995 ▶; Sheldrick, 2008 ▶) were also applied. Both cyclo­penta­dienyl rings were generated by using the AFIX 56 command. For atom pair C9/C9′, a further EADP instruction was applied to achieve reasonable anisotropic displacement ellipsoids.

## Supplementary Material

Crystal structure: contains datablock(s) I, new_global_publ_block. DOI: 10.1107/S1600536814020674/wm5048sup1.cif

Structure factors: contains datablock(s) I. DOI: 10.1107/S1600536814020674/wm5048Isup2.hkl

Supporting information file. DOI: 10.1107/S1600536814020674/wm5048Isup3.png

CCDC reference: 1018554

Additional supporting information:  crystallographic information; 3D view; checkCIF report

## Figures and Tables

**Table 1:** *T*-shaped π–π inter­action geometries (Å, °) for (I)

*D*⋯*D*	*D*⋯*D*	α^(i)^
C_6_H_3_(CF_3_)_2_⋯C_4_H_2_BrS^(ii)^	3.695 (4)	8.8 (3)
C_4_H_2_BrS⋯C_5_H_4_^(iii)^	4.943 (4)	88.3 (3)
C_4_H_2_BrS^(iv)^⋯C_5_H_4_^(iii)^	4.688 (6)	86.8 (5)

*D* denotes the centroids of the respective aromatic rings. (i) The angle α is described by the inter­section of the involved aromatics. (ii) Intra­molecular inter­action. (iii) Inter­molecular inter­action with symmetry code: –*x* + 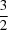, *y* – 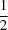, –*z* + 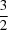. (iv): Disordered (′-labeled) part.

**Table 2:** Experimental details

Crystal data	
Chemical formula	[Fe(C_9_H_6_BrS)(C_13_H_7_F_6_)]
*M*_r_	559.14
Crystal system, space group	Monoclinic, *C*2/*c*
Temperature (K)	110
*a*, *b*, *c* (Å)	18.056 (5), 10.294 (5), 21.451 (5)
β (°)	93.268 (5)
*V* (Å^3^)	3981 (2)
*Z*	8
Radiation type	Mo *K*α
μ (mm^−1^)	2.93
Crystal size (mm)	0.4 × 0.4 × 0.2

Data collection	
Diffractometer	Oxford Gemini CCD
Absorption correction	Multi-scan (*CrysAlis RED*; Oxford Diffraction, 2006 ▶)
*T*_min_, *T*_max_	0.436, 1.000
No. of measured, independent and observed [*I* > 2σ(*I*)] reflections	11002, 3687, 2887
*R*_int_	0.035
(sin θ/λ)_max_ (Å^−1^)	0.606

Refinement	
*R*[*F* ^2^ > 2σ(*F* ^2^)], *wR*(*F* ^2^), *S*	0.044, 0.125, 1.00
No. of reflections	3687
No. of parameters	357
No. of restraints	258
H-atom treatment	H-atom parameters constrained
Δρ_max_, Δρ_min_ (e Å^−3^)	0.94, −0.61

Computer programs: *CrysAlis CCD* and *CrysAlis RED* (Oxford Diffraction, 2006 ▶), *SHELXS2013*, *SHELXL2013* and *SHELXTL*(Sheldrick, 2008 ▶), *ORTEP-3 for Windows* and *WinGX* (Farrugia, 2012 ▶) and *publCIF* (Westrip, 2010 ▶).

## supplementary crystallographic information

### Crystal data

**Table:**

[Fe(C_9_H_6_BrS)(C_13_H_7_F_6_)]	*F*(000) = 2208
*M**_r_* = 559.14	*D*_x_ = 1.866 Mg m^−^^3^
Monoclinic, *C*2/*c*	Mo *K*α radiation, λ = 0.71073 Å
*a* = 18.056 (5) Å	Cell parameters from 3003 reflections
*b* = 10.294 (5) Å	θ = 3.8–27.2°
*c* = 21.451 (5) Å	µ = 2.93 mm^−^^1^
β = 93.268 (5)°	*T* = 110 K
*V* = 3981 (2) Å^3^	Block, orange
*Z* = 8	0.4 × 0.4 × 0.2 mm

### Data collection

**Table:**

Oxford Gemini CCD diffractometer	2887 reflections with *I* > 2σ(*I*)
ω scans	*R*_int_ = 0.035
Absorption correction: multi-scan (*CrysAlis RED*; Oxford Diffraction, 2006)	θ_max_ = 25.5°, θ_min_ = 2.9°
*T*_min_ = 0.436, *T*_max_ = 1.000	*h* = −21→21
11002 measured reflections	*k* = −12→12
3687 independent reflections	*l* = −23→25

### Refinement

**Table:**

Refinement on *F*^2^	258 restraints
Least-squares matrix: full	Hydrogen site location: inferred from neighbouring sites
*R*[*F*^2^ > 2σ(*F*^2^)] = 0.044	H-atom parameters constrained
*wR*(*F*^2^) = 0.125	*w* = 1/[σ^2^(*F*_o_^2^) + (0.0737*P*)^2^ + 7.0529*P*] where *P* = (*F*_o_^2^ + 2*F*_c_^2^)/3
*S* = 1.00	(Δ/σ)_max_ = 0.004
3687 reflections	Δρ_max_ = 0.94 e Å^−^^3^
357 parameters	Δρ_min_ = −0.61 e Å^−^^3^

### Special details

**Table:**

Geometry. All e.s.d.'s (except the e.s.d. in the dihedral angle between two l.s. planes) are estimated using the full covariance matrix. The cell e.s.d.'s are taken into account individually in the estimation of e.s.d.'s in distances, angles and torsion angles; correlations between e.s.d.'s in cell parameters are only used, when they are defined by crystal symmetry. An approximate (isotropic) treatment of cell e.s.d.'s is used for estimating e.s.d.'s involving l.s. planes.

### Fractional atomic coordinates and isotropic or equivalent isotropic displacement parameters (Å^2^)

**Table:**

	*x*	*y*	*z*	*U*_iso_*/*U*_eq_	Occ. (<1)
S1	0.59952 (12)	−0.21423 (19)	0.60297 (8)	0.0419 (4)	0.6
Br1	0.63183 (5)	0.10861 (8)	0.73209 (4)	0.0409 (2)	0.6
C1	0.6910 (4)	−0.2085 (6)	0.6296 (3)	0.0323 (13)	0.6
H1	0.7272	−0.2654	0.6168	0.039*	0.6
C2	0.7045 (3)	−0.1088 (5)	0.6724 (3)	0.0259 (11)	0.6
C3	0.6392 (4)	−0.0394 (6)	0.6811 (3)	0.0297 (13)	0.6
C4	0.5776 (5)	−0.0824 (8)	0.6470 (3)	0.0342 (17)	0.6
H4	0.5307	−0.0454	0.6477	0.041*	0.6
C5	0.7792 (3)	−0.0860 (8)	0.7008 (3)	0.0274 (17)	0.6
C6	0.7990 (4)	−0.0156 (9)	0.7562 (3)	0.034 (2)	0.6
H6	0.7667	0.0301	0.7804	0.041*	0.6
C7	0.8769 (4)	−0.0272 (8)	0.7683 (3)	0.037 (2)	0.6
H7	0.9045	0.0095	0.8017	0.045*	0.6
C8	0.9052 (3)	−0.1047 (6)	0.7204 (3)	0.0367 (18)	0.6
H8	0.9546	−0.1277	0.7169	0.044*	0.6
C9	0.8449 (3)	−0.1410 (6)	0.6786 (2)	0.0303 (14)	0.6
H9	0.8478	−0.1920	0.6431	0.036*	0.6
S1'	0.56009 (19)	0.0774 (3)	0.7152 (2)	0.0816 (12)	0.4
Br1'	0.64769 (11)	−0.25200 (13)	0.61634 (6)	0.0613 (4)	0.4
C1'	0.6502 (7)	0.0577 (13)	0.7354 (8)	0.063 (4)	0.4
H1'	0.6753	0.1051	0.7669	0.076*	0.4
C2'	0.6844 (6)	−0.0381 (9)	0.6999 (5)	0.037 (2)	0.4
C3'	0.6298 (7)	−0.1011 (10)	0.6644 (5)	0.046 (3)	0.4
C4'	0.5597 (7)	−0.0537 (14)	0.6654 (7)	0.060 (4)	0.4
H4'	0.5183	−0.0866	0.6429	0.072*	0.4
C5'	0.7644 (4)	−0.0653 (12)	0.7128 (5)	0.030 (3)	0.4
C6'	0.8083 (7)	−0.0167 (14)	0.7647 (5)	0.035 (3)	0.4
H6'	0.7916	0.0346	0.7967	0.042*	0.4
C7'	0.8823 (5)	−0.0602 (13)	0.7592 (5)	0.040 (3)	0.4
H7'	0.9225	−0.0424	0.7869	0.047*	0.4
C8'	0.8841 (5)	−0.1356 (10)	0.7039 (5)	0.039 (3)	0.4
H8'	0.9257	−0.1759	0.6890	0.047*	0.4
C9'	0.8113 (5)	−0.1388 (9)	0.6752 (4)	0.0303 (14)	0.4
H9'	0.7968	−0.1814	0.6383	0.036*	0.4
C10	0.81000 (19)	0.1698 (3)	0.61277 (16)	0.0300 (8)	
C11	0.8794 (2)	0.1132 (4)	0.59760 (18)	0.0387 (9)	
H11	0.8870	0.0601	0.5635	0.046*	
C12	0.9345 (2)	0.1522 (4)	0.6438 (2)	0.0467 (10)	
H12	0.9844	0.1291	0.6451	0.056*	
C13	0.9005 (2)	0.2321 (4)	0.6870 (2)	0.0442 (10)	
H13	0.9241	0.2712	0.7218	0.053*	
C14	0.8247 (2)	0.2427 (3)	0.66885 (18)	0.0345 (8)	
H14	0.7898	0.2896	0.6899	0.041*	
C15	0.73864 (19)	0.1567 (3)	0.57697 (14)	0.0271 (7)	
C16	0.7274 (2)	0.0608 (3)	0.53160 (15)	0.0311 (8)	
H16	0.7651	0.0018	0.5247	0.037*	
C17	0.6609 (2)	0.0524 (3)	0.49668 (16)	0.0356 (9)	
C18	0.6033 (2)	0.1366 (4)	0.50595 (16)	0.0345 (8)	
H18	0.5585	0.1296	0.4825	0.041*	
C19	0.6138 (2)	0.2321 (4)	0.55113 (15)	0.0318 (8)	
C20	0.6805 (2)	0.2420 (3)	0.58615 (15)	0.0299 (8)	
H20	0.6865	0.3067	0.6163	0.036*	
C21	0.6527 (3)	−0.0494 (4)	0.44666 (19)	0.0481 (11)	
C22	0.5538 (2)	0.3269 (4)	0.56256 (17)	0.0434 (10)	
F1	0.67577 (17)	−0.1660 (2)	0.46565 (11)	0.0628 (8)	
F2	0.6945 (2)	−0.0225 (3)	0.39867 (11)	0.0786 (10)	
F3	0.5850 (2)	−0.0629 (4)	0.42356 (19)	0.1139 (16)	
F4	0.52097 (15)	0.3028 (3)	0.61561 (11)	0.0669 (8)	
F5	0.50019 (14)	0.3292 (3)	0.51730 (11)	0.0582 (7)	
F6	0.57912 (16)	0.4492 (2)	0.56767 (14)	0.0655 (8)	
Fe1	0.85187 (3)	0.05282 (5)	0.68322 (2)	0.02830 (18)	

### Atomic displacement parameters (Å^2^)

**Table:**

	*U*^11^	*U*^22^	*U*^33^	*U*^12^	*U*^13^	*U*^23^
S1	0.0414 (11)	0.0476 (11)	0.0357 (9)	−0.0166 (9)	−0.0064 (8)	−0.0030 (8)
Br1	0.0436 (5)	0.0359 (4)	0.0437 (4)	0.0049 (3)	0.0063 (3)	−0.0053 (3)
C1	0.025 (3)	0.029 (3)	0.042 (3)	−0.017 (3)	−0.007 (3)	0.003 (2)
C2	0.029 (3)	0.022 (2)	0.028 (2)	−0.005 (2)	0.005 (2)	0.002 (2)
C3	0.031 (3)	0.029 (3)	0.030 (3)	−0.004 (3)	0.002 (3)	0.003 (3)
C4	0.028 (4)	0.039 (4)	0.036 (4)	−0.004 (3)	0.005 (3)	0.007 (3)
C5	0.033 (3)	0.029 (3)	0.020 (3)	−0.005 (3)	0.001 (3)	0.001 (3)
C6	0.038 (4)	0.043 (4)	0.021 (3)	−0.008 (3)	−0.002 (3)	0.001 (3)
C7	0.039 (4)	0.037 (4)	0.035 (3)	−0.007 (3)	−0.009 (3)	0.002 (3)
C8	0.039 (4)	0.029 (3)	0.040 (4)	0.001 (3)	−0.011 (3)	−0.003 (3)
C9	0.024 (4)	0.0264 (19)	0.039 (2)	−0.003 (3)	−0.005 (3)	0.0004 (18)
S1'	0.0422 (18)	0.0499 (17)	0.156 (4)	0.0105 (14)	0.037 (2)	0.023 (2)
Br1'	0.0844 (11)	0.0503 (8)	0.0479 (7)	−0.0304 (8)	−0.0074 (7)	−0.0028 (5)
C1'	0.062 (7)	0.044 (6)	0.085 (7)	0.010 (5)	0.007 (5)	0.006 (6)
C2'	0.033 (4)	0.032 (4)	0.048 (4)	−0.002 (4)	0.006 (4)	0.011 (4)
C3'	0.039 (5)	0.043 (5)	0.056 (5)	−0.013 (4)	−0.009 (4)	0.020 (4)
C4'	0.043 (6)	0.071 (7)	0.064 (7)	−0.018 (5)	−0.001 (5)	0.028 (5)
C5'	0.028 (4)	0.031 (5)	0.032 (5)	−0.010 (4)	0.010 (4)	0.007 (4)
C6'	0.035 (5)	0.044 (5)	0.026 (5)	−0.007 (4)	0.005 (4)	0.006 (4)
C7'	0.033 (5)	0.047 (6)	0.039 (5)	0.003 (4)	0.004 (4)	0.011 (4)
C8'	0.038 (6)	0.039 (5)	0.042 (6)	0.007 (5)	0.013 (5)	0.007 (5)
C9'	0.024 (4)	0.0264 (19)	0.039 (2)	−0.003 (3)	−0.005 (3)	0.0004 (18)
C10	0.031 (2)	0.0257 (17)	0.0336 (18)	−0.0006 (14)	0.0084 (15)	0.0061 (14)
C11	0.039 (2)	0.042 (2)	0.036 (2)	0.0058 (18)	0.0173 (17)	0.0097 (17)
C12	0.028 (2)	0.051 (2)	0.062 (3)	−0.0058 (19)	0.0121 (19)	0.012 (2)
C13	0.030 (2)	0.038 (2)	0.064 (3)	−0.0095 (17)	−0.0013 (19)	0.0023 (19)
C14	0.033 (2)	0.0271 (18)	0.043 (2)	−0.0035 (15)	0.0017 (16)	0.0000 (15)
C15	0.0324 (19)	0.0267 (17)	0.0229 (16)	0.0003 (15)	0.0074 (14)	0.0068 (13)
C16	0.041 (2)	0.0266 (18)	0.0267 (17)	0.0064 (15)	0.0072 (15)	0.0029 (14)
C17	0.054 (3)	0.0280 (18)	0.0248 (17)	0.0008 (17)	0.0014 (17)	−0.0025 (14)
C18	0.041 (2)	0.0336 (19)	0.0281 (18)	0.0007 (16)	−0.0017 (16)	−0.0015 (15)
C19	0.036 (2)	0.0347 (19)	0.0249 (17)	0.0057 (16)	0.0043 (15)	0.0016 (14)
C20	0.040 (2)	0.0271 (17)	0.0235 (17)	0.0034 (15)	0.0065 (15)	−0.0001 (13)
C21	0.069 (3)	0.037 (2)	0.038 (2)	0.006 (2)	−0.002 (2)	−0.0067 (18)
C22	0.043 (2)	0.055 (3)	0.031 (2)	0.0147 (19)	−0.0054 (18)	−0.0054 (18)
F1	0.116 (2)	0.0309 (13)	0.0419 (13)	0.0021 (13)	0.0113 (14)	−0.0070 (10)
F2	0.161 (3)	0.0460 (15)	0.0316 (13)	0.0019 (17)	0.0266 (16)	−0.0061 (11)
F3	0.087 (3)	0.115 (3)	0.133 (3)	0.034 (2)	−0.053 (2)	−0.093 (3)
F4	0.0594 (17)	0.101 (2)	0.0415 (14)	0.0347 (16)	0.0151 (12)	−0.0019 (14)
F5	0.0474 (15)	0.0777 (18)	0.0474 (14)	0.0252 (13)	−0.0145 (11)	−0.0153 (13)
F6	0.0636 (18)	0.0427 (15)	0.088 (2)	0.0220 (13)	−0.0152 (15)	−0.0183 (13)
Fe1	0.0262 (3)	0.0287 (3)	0.0302 (3)	−0.0004 (2)	0.0033 (2)	0.0008 (2)

### Geometric parameters (Å, º)

**Table:**

S1—C1	1.717 (6)	C7'—Fe1	2.052 (12)
S1—C4	1.713 (8)	C7'—H7'	0.9300
Br1—C3	1.885 (7)	C8'—C9'	1.4200
C1—C2	1.389 (8)	C8'—Fe1	2.066 (9)
C1—H1	0.9300	C8'—H8'	0.9300
C2—C3	1.399 (8)	C9'—Fe1	2.107 (9)
C2—C5	1.469 (7)	C9'—H9'	0.9300
C3—C4	1.370 (10)	C10—C14	1.430 (5)
C4—H4	0.9300	C10—C11	1.436 (5)
C5—C9	1.4200	C10—C15	1.468 (5)
C5—C6	1.4200	C10—Fe1	2.043 (3)
C5—Fe1	1.990 (8)	C11—C12	1.422 (6)
C6—C7	1.4200	C11—Fe1	2.027 (4)
C6—Fe1	2.007 (9)	C11—H11	0.9300
C6—H6	0.9300	C12—C13	1.407 (6)
C7—C8	1.4200	C12—Fe1	2.033 (4)
C7—Fe1	2.029 (8)	C12—H12	0.9300
C7—H7	0.9300	C13—C14	1.406 (5)
C8—C9	1.4200	C13—Fe1	2.043 (4)
C8—Fe1	2.026 (6)	C13—H13	0.9300
C8—H8	0.9300	C14—Fe1	2.035 (4)
C9—Fe1	2.002 (6)	C14—H14	0.9300
C9—H9	0.9300	C15—C16	1.392 (5)
S1'—C1'	1.672 (13)	C15—C20	1.391 (5)
S1'—C4'	1.721 (13)	C16—C17	1.381 (5)
Br1'—C3'	1.902 (11)	C16—H16	0.9300
C1'—C2'	1.410 (14)	C17—C18	1.377 (5)
C1'—H1'	0.9300	C17—C21	1.501 (5)
C2'—C3'	1.373 (13)	C18—C19	1.386 (5)
C2'—C5'	1.483 (13)	C18—H18	0.9300
C3'—C4'	1.359 (14)	C19—C20	1.386 (5)
C4'—H4'	0.9300	C19—C22	1.489 (5)
C5'—C6'	1.4200	C20—H20	0.9300
C5'—C9'	1.4200	C21—F3	1.300 (6)
C5'—Fe1	2.118 (11)	C21—F1	1.327 (5)
C6'—C7'	1.4200	C21—F2	1.339 (5)
C6'—Fe1	2.084 (14)	C22—F5	1.332 (4)
C6'—H6'	0.9300	C22—F4	1.336 (5)
C7'—C8'	1.4200	C22—F6	1.341 (5)
			
C1—S1—C4	92.1 (4)	C13—C12—H12	126.0
C2—C1—S1	112.0 (5)	C11—C12—H12	126.0
C2—C1—H1	124.0	Fe1—C12—H12	126.1
S1—C1—H1	124.0	C12—C13—C14	108.5 (4)
C1—C2—C3	110.4 (6)	C12—C13—Fe1	69.4 (2)
C1—C2—C5	121.0 (6)	C14—C13—Fe1	69.5 (2)
C3—C2—C5	128.5 (6)	C12—C13—H13	125.8
C4—C3—C2	115.3 (6)	C14—C13—H13	125.8
C4—C3—Br1	119.3 (5)	Fe1—C13—H13	126.9
C2—C3—Br1	125.4 (5)	C13—C14—C10	108.9 (3)
C3—C4—S1	110.2 (6)	C13—C14—Fe1	70.1 (2)
C3—C4—H4	124.9	C10—C14—Fe1	69.8 (2)
S1—C4—H4	124.9	C13—C14—H14	125.6
C9—C5—C6	108.0	C10—C14—H14	125.6
C9—C5—C2	124.2 (5)	Fe1—C14—H14	126.1
C6—C5—C2	127.7 (5)	C16—C15—C20	117.7 (3)
C9—C5—Fe1	69.6 (3)	C16—C15—C10	121.3 (3)
C6—C5—Fe1	69.8 (3)	C20—C15—C10	121.0 (3)
C2—C5—Fe1	129.5 (5)	C17—C16—C15	120.7 (3)
C7—C6—C5	108.0	C17—C16—H16	119.6
C7—C6—Fe1	70.3 (3)	C15—C16—H16	119.6
C5—C6—Fe1	68.5 (3)	C18—C17—C16	121.5 (3)
C7—C6—H6	126.0	C18—C17—C21	119.9 (4)
C5—C6—H6	126.0	C16—C17—C21	118.6 (4)
Fe1—C6—H6	126.8	C17—C18—C19	118.2 (4)
C6—C7—C8	108.0	C17—C18—H18	120.9
C6—C7—Fe1	68.6 (3)	C19—C18—H18	120.9
C8—C7—Fe1	69.4 (3)	C18—C19—C20	120.8 (3)
C6—C7—H7	126.0	C18—C19—C22	120.5 (3)
C8—C7—H7	126.0	C20—C19—C22	118.7 (3)
Fe1—C7—H7	127.6	C19—C20—C15	121.1 (3)
C9—C8—C7	108.0	C19—C20—H20	119.5
C9—C8—Fe1	68.4 (3)	C15—C20—H20	119.5
C7—C8—Fe1	69.6 (3)	F3—C21—F1	107.0 (4)
C9—C8—H8	126.0	F3—C21—F2	106.7 (4)
C7—C8—H8	126.0	F1—C21—F2	104.0 (3)
Fe1—C8—H8	127.5	F3—C21—C17	113.5 (4)
C8—C9—C5	108.0	F1—C21—C17	113.3 (3)
C8—C9—Fe1	70.3 (3)	F2—C21—C17	111.6 (4)
C5—C9—Fe1	68.7 (3)	F5—C22—F4	106.5 (3)
C8—C9—H9	126.0	F5—C22—F6	105.9 (3)
C5—C9—H9	126.0	F4—C22—F6	105.7 (3)
Fe1—C9—H9	126.6	F5—C22—C19	113.4 (3)
C1'—S1'—C4'	92.0 (7)	F4—C22—C19	112.4 (3)
C2'—C1'—S1'	113.2 (11)	F6—C22—C19	112.4 (4)
C2'—C1'—H1'	123.4	C5—Fe1—C9	41.68 (12)
S1'—C1'—H1'	123.4	C5—Fe1—C6	41.62 (17)
C3'—C2'—C1'	107.9 (11)	C9—Fe1—C6	69.9 (2)
C3'—C2'—C5'	132.5 (10)	C5—Fe1—C11	126.2 (2)
C1'—C2'—C5'	118.8 (10)	C9—Fe1—C11	106.21 (19)
C4'—C3'—C2'	117.5 (11)	C6—Fe1—C11	165.1 (2)
C4'—C3'—Br1'	119.3 (9)	C5—Fe1—C8	69.79 (16)
C2'—C3'—Br1'	123.2 (8)	C9—Fe1—C8	41.28 (10)
C3'—C4'—S1'	108.7 (10)	C6—Fe1—C8	69.5 (2)
C3'—C4'—H4'	125.6	C11—Fe1—C8	117.8 (2)
S1'—C4'—H4'	125.6	C5—Fe1—C7	69.7 (2)
C6'—C5'—C9'	108.0	C9—Fe1—C7	69.49 (16)
C6'—C5'—C2'	125.1 (9)	C6—Fe1—C7	41.19 (17)
C9'—C5'—C2'	126.8 (9)	C11—Fe1—C7	152.3 (2)
C6'—C5'—Fe1	69.0 (4)	C8—Fe1—C7	40.99 (13)
C9'—C5'—Fe1	70.0 (4)	C5—Fe1—C14	124.2 (2)
C2'—C5'—Fe1	124.8 (8)	C9—Fe1—C14	159.5 (2)
C5'—C6'—C7'	108.0	C6—Fe1—C14	109.5 (2)
C5'—C6'—Fe1	71.5 (4)	C11—Fe1—C14	68.76 (15)
C7'—C6'—Fe1	68.7 (4)	C8—Fe1—C14	159.0 (2)
C5'—C6'—H6'	126.0	C7—Fe1—C14	124.4 (2)
C7'—C6'—H6'	126.0	C5—Fe1—C12	161.4 (2)
Fe1—C6'—H6'	125.3	C9—Fe1—C12	121.8 (2)
C8'—C7'—C6'	108.0	C6—Fe1—C12	153.4 (2)
C8'—C7'—Fe1	70.4 (4)	C11—Fe1—C12	40.99 (16)
C6'—C7'—Fe1	71.2 (4)	C8—Fe1—C12	102.9 (2)
C8'—C7'—H7'	126.0	C7—Fe1—C12	116.6 (3)
C6'—C7'—H7'	126.0	C14—Fe1—C12	68.27 (17)
Fe1—C7'—H7'	124.1	C5—Fe1—C13	158.0 (2)
C9'—C8'—C7'	108.0	C9—Fe1—C13	158.2 (2)
C9'—C8'—Fe1	71.7 (4)	C6—Fe1—C13	120.5 (2)
C7'—C8'—Fe1	69.3 (5)	C11—Fe1—C13	68.45 (17)
C9'—C8'—H8'	126.0	C8—Fe1—C13	120.9 (2)
C7'—C8'—H8'	126.0	C7—Fe1—C13	104.9 (2)
Fe1—C8'—H8'	124.6	C14—Fe1—C13	40.33 (15)
C8'—C9'—C5'	108.0	C12—Fe1—C13	40.38 (18)
C8'—C9'—Fe1	68.6 (4)	C5—Fe1—C10	110.1 (2)
C5'—C9'—Fe1	70.8 (4)	C9—Fe1—C10	122.11 (19)
C8'—C9'—H9'	126.0	C6—Fe1—C10	127.7 (2)
C5'—C9'—H9'	126.0	C11—Fe1—C10	41.30 (14)
Fe1—C9'—H9'	126.2	C8—Fe1—C10	155.5 (2)
C14—C10—C11	106.3 (3)	C7—Fe1—C10	163.4 (2)
C14—C10—C15	127.1 (3)	C14—Fe1—C10	41.06 (14)
C11—C10—C15	126.5 (3)	C12—Fe1—C10	69.21 (16)
C14—C10—Fe1	69.2 (2)	C13—Fe1—C10	68.75 (16)
C11—C10—Fe1	68.8 (2)	C11—Fe1—C7'	145.6 (3)
C15—C10—Fe1	127.6 (2)	C14—Fe1—C7'	135.8 (3)
C12—C11—C10	108.2 (3)	C12—Fe1—C7'	116.5 (3)
C12—C11—Fe1	69.7 (2)	C13—Fe1—C7'	112.7 (3)
C10—C11—Fe1	69.93 (19)	C10—Fe1—C7'	173.1 (3)
C12—C11—H11	125.9	C11—Fe1—C8'	113.7 (3)
C10—C11—H11	125.9	C14—Fe1—C8'	175.6 (3)
Fe1—C11—H11	126.0	C12—Fe1—C8'	110.9 (3)
C13—C12—C11	108.1 (4)	C13—Fe1—C8'	136.4 (3)
C13—C12—Fe1	70.2 (2)	C10—Fe1—C8'	143.1 (3)
C11—C12—Fe1	69.3 (2)	C7'—Fe1—C8'	40.34 (19)
			
C4—S1—C1—C2	1.1 (5)	Fe1—C7'—C8'—C9'	−61.6 (4)
S1—C1—C2—C3	−0.8 (6)	C6'—C7'—C8'—Fe1	61.6 (4)
S1—C1—C2—C5	−179.7 (5)	C7'—C8'—C9'—C5'	0.0
C1—C2—C3—C4	0.1 (8)	Fe1—C8'—C9'—C5'	−60.0 (5)
C5—C2—C3—C4	178.8 (7)	C7'—C8'—C9'—Fe1	60.0 (5)
C1—C2—C3—Br1	−177.0 (4)	C6'—C5'—C9'—C8'	0.0
C5—C2—C3—Br1	1.7 (9)	C2'—C5'—C9'—C8'	177.7 (12)
C2—C3—C4—S1	0.7 (8)	Fe1—C5'—C9'—C8'	58.6 (4)
Br1—C3—C4—S1	178.0 (4)	C6'—C5'—C9'—Fe1	−58.6 (4)
C1—S1—C4—C3	−1.0 (6)	C2'—C5'—C9'—Fe1	119.1 (12)
C1—C2—C5—C9	14.0 (10)	C14—C10—C11—C12	−0.2 (4)
C3—C2—C5—C9	−164.6 (6)	C15—C10—C11—C12	178.8 (3)
C1—C2—C5—C6	−161.0 (6)	Fe1—C10—C11—C12	−59.4 (3)
C3—C2—C5—C6	20.4 (10)	C14—C10—C11—Fe1	59.2 (2)
C1—C2—C5—Fe1	104.9 (7)	C15—C10—C11—Fe1	−121.7 (3)
C3—C2—C5—Fe1	−73.8 (8)	C10—C11—C12—C13	−0.1 (4)
C9—C5—C6—C7	0.0	Fe1—C11—C12—C13	−59.7 (3)
C2—C5—C6—C7	175.7 (8)	C10—C11—C12—Fe1	59.6 (2)
Fe1—C5—C6—C7	−59.4 (3)	C11—C12—C13—C14	0.4 (5)
C9—C5—C6—Fe1	59.4 (3)	Fe1—C12—C13—C14	−58.7 (3)
C2—C5—C6—Fe1	−125.0 (7)	C11—C12—C13—Fe1	59.1 (3)
C5—C6—C7—C8	0.0	C12—C13—C14—C10	−0.6 (4)
Fe1—C6—C7—C8	−58.3 (2)	Fe1—C13—C14—C10	−59.2 (2)
C5—C6—C7—Fe1	58.3 (2)	C12—C13—C14—Fe1	58.6 (3)
C6—C7—C8—C9	0.0	C11—C10—C14—C13	0.5 (4)
Fe1—C7—C8—C9	−57.8 (3)	C15—C10—C14—C13	−178.6 (3)
C6—C7—C8—Fe1	57.8 (3)	Fe1—C10—C14—C13	59.4 (3)
C7—C8—C9—C5	0.0	C11—C10—C14—Fe1	−59.0 (2)
Fe1—C8—C9—C5	−58.5 (3)	C15—C10—C14—Fe1	122.0 (3)
C7—C8—C9—Fe1	58.5 (3)	C14—C10—C15—C16	−165.3 (3)
C6—C5—C9—C8	0.0	C11—C10—C15—C16	15.8 (5)
C2—C5—C9—C8	−175.9 (8)	Fe1—C10—C15—C16	−74.2 (4)
Fe1—C5—C9—C8	59.5 (3)	C14—C10—C15—C20	16.3 (5)
C6—C5—C9—Fe1	−59.5 (3)	C11—C10—C15—C20	−162.5 (3)
C2—C5—C9—Fe1	124.6 (7)	Fe1—C10—C15—C20	107.4 (3)
C4'—S1'—C1'—C2'	7.1 (12)	C20—C15—C16—C17	0.7 (5)
S1'—C1'—C2'—C3'	−8.6 (14)	C10—C15—C16—C17	−177.7 (3)
S1'—C1'—C2'—C5'	179.8 (9)	C15—C16—C17—C18	−1.0 (5)
C1'—C2'—C3'—C4'	6.2 (16)	C15—C16—C17—C21	177.6 (3)
C5'—C2'—C3'—C4'	176.2 (12)	C16—C17—C18—C19	0.7 (5)
C1'—C2'—C3'—Br1'	−172.1 (9)	C21—C17—C18—C19	−177.9 (3)
C5'—C2'—C3'—Br1'	−2.1 (17)	C17—C18—C19—C20	−0.2 (5)
C2'—C3'—C4'—S1'	−1.2 (15)	C17—C18—C19—C22	179.2 (3)
Br1'—C3'—C4'—S1'	177.2 (6)	C18—C19—C20—C15	0.0 (5)
C1'—S1'—C4'—C3'	−3.4 (12)	C22—C19—C20—C15	−179.4 (3)
C3'—C2'—C5'—C6'	−159.0 (11)	C16—C15—C20—C19	−0.2 (5)
C1'—C2'—C5'—C6'	10.1 (15)	C10—C15—C20—C19	178.1 (3)
C3'—C2'—C5'—C9'	23.6 (18)	C18—C17—C21—F3	−11.8 (6)
C1'—C2'—C5'—C9'	−167.3 (11)	C16—C17—C21—F3	169.6 (4)
C3'—C2'—C5'—Fe1	113.6 (12)	C18—C17—C21—F1	−134.1 (4)
C1'—C2'—C5'—Fe1	−77.3 (13)	C16—C17—C21—F1	47.3 (5)
C9'—C5'—C6'—C7'	0.0	C18—C17—C21—F2	108.9 (5)
C2'—C5'—C6'—C7'	−177.8 (12)	C16—C17—C21—F2	−69.7 (5)
Fe1—C5'—C6'—C7'	−59.3 (4)	C18—C19—C22—F5	−14.8 (5)
C9'—C5'—C6'—Fe1	59.3 (4)	C20—C19—C22—F5	164.6 (3)
C2'—C5'—C6'—Fe1	−118.5 (10)	C18—C19—C22—F4	106.1 (4)
C5'—C6'—C7'—C8'	0.0	C20—C19—C22—F4	−74.5 (5)
Fe1—C6'—C7'—C8'	−61.1 (4)	C18—C19—C22—F6	−134.8 (4)
C5'—C6'—C7'—Fe1	61.1 (4)	C20—C19—C22—F6	44.6 (5)
C6'—C7'—C8'—C9'	0.0		
