# Supplementary figures and images for: Crystal structure of 3-{1′-[3,5-bis­(tri­fluoro­meth­yl)phen­yl]ferrocenyl}-4-bromo­thio­phene
Source: Acta Crystallogr Sect E Struct Rep Online. 2014 Sep 24;70(Pt 10):238–41. doi: 10.1107/S1600536814020674 (PMC4257188; doi:10.1107/S1600536814020674)

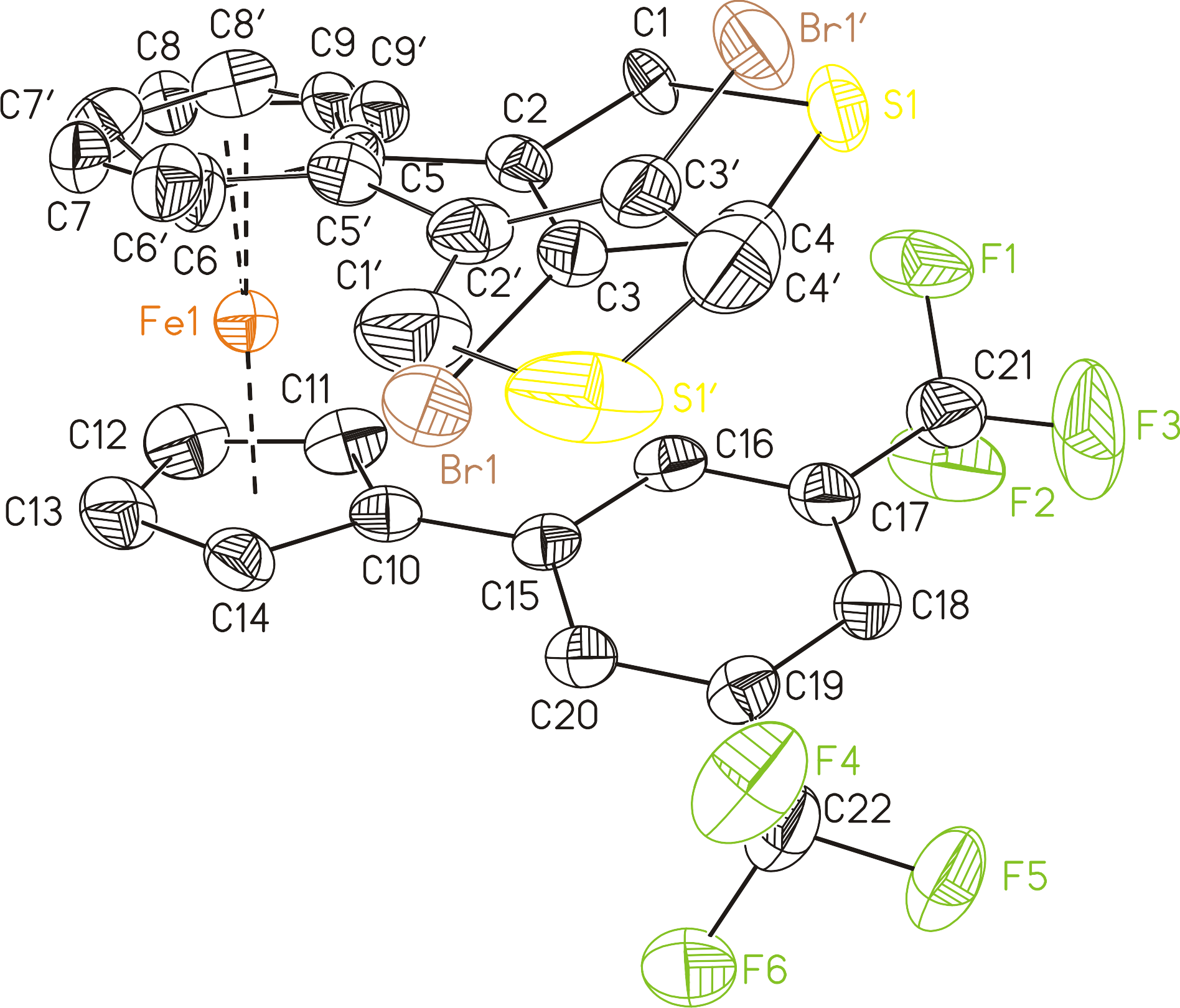

Supplement: Supplementary file 3 [file e-70-00238-Isup3.png]
